# Nurses’ assessed self-efficacy levels to medical asepsis and their relation to structural empowerment, work engagement and work-related stress

**DOI:** 10.3233/WOR-211305

**Published:** 2023-02-14

**Authors:** Lisa Arvidsson, Bernice Skytt, Maria Lindberg, Magnus Lindberg

**Affiliations:** a Department of Caring Sciences, Faculty of Health and Occupational Studies, University of Gävle, Gävle, Sweden; b Department of Public Health and Caring Sciences, Uppsala University, Uppsala, Sweden; c Centre for Research and Development, Uppsala University/County Council of Gävleborg, Gävle, Sweden

**Keywords:** Infection prevention, working conditions, registered nurses, licensed practical nurses

## Abstract

**BACKGROUND:**

Nurses’ working conditions are important for their well-being at work and for their ability to provide patients with safe care. Self-efficacy can influence employees’ behaviour at work. Therefore, it is valuable to study self-efficacy levels to medical asepsis in relation to working conditions.

**OBJECTIVE:**

To investigate the relationship between nurses’ assessed self-efficacy levels to medical asepsis in care situations and structural empowerment, work engagement and work-related stress.

**METHODS:**

A cross-sectional study with a correlational design was conducted. A total of 417 registered nurses and licensed practical nurses at surgical and orthopaedic units responded to a questionnaire containing: the Infection Prevention Appraisal Scale, the Conditions of Work Effectiveness Questionnaire-II, the Utrecht Work Engagement Scale-9 and the Health & Safety Executive Management Standards Indicator Tool. Correlational analyses and group comparisons were performed.

**RESULTS:**

The nurses rated high levels of self-efficacy to medical asepsis in care situations. The correlational analyses revealed that correlation coefficients between structural empowerment, work engagement, work-related stress and self-efficacy to medical asepsis were 0.254–0.268. Significant differences in self-efficacy were found in the grouped working conditions.

**CONCLUSIONS:**

This study revealed that nurses rated high self-efficacy levels to medical asepsis and, to some extent, this seemed related to structural empowerment, work engagement and work-related stress. This valuable knowledge could enable improvements at the managerial and organisational levels, benefiting both nurses and patients in the long run.

## 1 Introduction

The working conditions of nurses are related to their well-being and satisfaction at work, and undesirable working conditions lead to a higher risk of dissatisfaction and intention to leave the profession [1–4]. Working conditions such as structural empowerment, work engagement and low work-related stress are also important to enable nurses to provide good, safe care to patients [5–7]. Medical asepsis in care situations involves procedures that reduce micro-organisms and decrease the risk for organism transmission in healthcare [8]. Organism transmission in healthcare can lead to healthcare-associated infections. The most frequently reported healthcare-associated infections are respiratory tract infections, urinary tract infections and surgical site infections [9]. Self-efficacy is described as a person’s belief in their ability to succeed in specified situations and has been shown to affect behaviour [10]. Human behaviour is known to be a consequence of a causal part of a sequence of events, affected by the context in which individuals are operating [11]. Accordingly, it is valuable to study the relationship between the assessed working conditions of nurses and self-efficacy to medical asepsis in care situations.

### 1.1 Self-efficacy

People with high self-efficacy are more able to take action and are more likely to view challenges as something to be handled rather than problems and things to avoid [10]. Individuals with high self-efficacy are more prone to making an effort to complete tasks and are more productive and creative than individuals with lower self-efficacy [12, 13]. Bandura described self-efficacy as based on four essential elements: 1, Past performance outcomes, which are indicators of capability. 2, Vicarious experiences, including observing others complete tasks successfully and the transmission of competencies. 3, Verbal persuasion, meaning that people are coached by others to believe they can complete tasks successfully. 4, Psychological/affective states which influence a person’s beliefs in their capabilities [10]. Self-efficacy has been found to influence employees’ motivation, perceptions, and performance at work [12]. A systematic review of systematic reviews investigated interventions to improve the hand hygiene behaviour of healthcare personnel and concluded that self-efficacy and social influence may enhance the effectiveness of interventions, but that literature regarding this was relatively scarce and more research was needed [14].

### 1.2 Working conditions

#### 1.2.1 Structural empowerment

According to Kanter, a work environment that provides employees with access to information, resources, support and opportunities is empowering. Good structural empowerment leads to organisational effectiveness and people feeling in control at work [15]. Access to *information* refers to people knowing the work and the organisation. Access to r*esources* involves employees’ ability to access sufficient time, materials and resources to achieve organisational goals. Access to *opportunity* describes the changes for professional development within the organisation. Access to s*upport* refers to obtaining guidance and feedback from managers, subordinates and peers. Access to these structures depends on perceptions of formal and informal power, where formal power means having a visible and central job that contributes to achieving organisational goals, and informal power is described as being developed through work-related alliances [15, 16]. A scoping review found that structural empowerment, especially sufficient access to support and resources, positively influenced work and unit effectiveness and affected the quality of care and patient safety climate [5]. Another systematic review of qualitative literature found that healthcare personnel’s perceptions of the work environment, e.g., access to resources and information, influenced compliance with hygiene principles and the authors concluded that healthcare personnel’s perceptions of their work environment were in line with Kanter’s theory of structural empowerment [17].

#### 1.2.2 Work engagement

Work engagement is described as ‘a positive, fulfilling work-related state of mind that is characterised by vigour, dedication and absorption’ (18 p. 74). Engagement is characterised as a persistent cognitive state not focusing on a particular object or event. Vigour means high levels of energy while working and a willingness to invest effort in work. Dedication is characterised by a sense of enthusiasm, meaning, pride and inspiration, and absorption is described as being fully concentrated and happily engrossed in work, leading to time passing quickly [19]. The construct of work engagement is used as an indicator of a healthy workplace [18] and high levels of work engagement has been reported to increase job satisfaction and the intention to remain in a profession [20].

#### 1.2.3 Work-related stress

Work-related stress has long been known as a common concern among healthcare personnel worldwide [21, 22], and has been found to make cognitive failure more likely and thus affect patient safety [23]. Work-related stress can encompass several stressor areas, such as demand, control, support, relationships, role and change [24]. Demands relate to workload, work patterns and working environment. Control refers to employee autonomy and how much say people have in their work. Support includes encouragement, and the dimension is further divided into two subscales: ‘Management support’ and ‘Colleague support’. Relationships involve how conflicts and unacceptable behaviours are addressed and how a positive working environment is promoted. Role refers to how well people understand their role within the organisation and whether the organisation ensures a person does not have several conflicting roles. Change measures how organisational changes are managed and communicated within the organisation [24].

In light of the above, working conditions for nurses are important for their well-being at work [1–3]. Structural empowerment, work engagement and low levels of work-related stress have also been found to be essential for enabling nurses’ provision of safe care to patients [5–7]. Nevertheless, working conditions for nurses are often reported as strained [4]. Self-efficacy refers to people’s beliefs in their ability to succeed in specified situations and has been found to influence employee performance, i.e., behaviours, at work [12]. Therefore, it is valuable to study nurses’ assessed self-efficacy levels to medical asepsis in relation to structural empowerment, work engagement and work-related stress, to enable appropriate implementation measures for nurses. Although this topic is well-researched, there are, to our knowledge, no previous studies focusing on nurses’ assessed self-efficacy levels to medical asepsis in care situations in relation to different working condition measurements, neither in correlational analyses nor in group comparisons.

### 1.3 Objective and hypothesis

The study aimed to investigate the relationship between nurses’ assessed self-efficacy levels to medical asepsis in care situations and structural empowerment, work engagement and work-related stress.

We hypothesised: **H1** Nurses who rate high levels of structural empowerment also rate high levels of self-efficacy to medical asepsis. **H2** Nurses who rate high levels of work engagement also rate high levels of self-efficacy to medical asepsis. **H3** Nurses who rate low levels of work-related stress also rate high levels of self-efficacy to medical asepsis. Further, we were interested in determining if the assessment of risk for organism transmission at work was related to self-efficacy to medical asepsis and an additional hypothesis therefore was generated. **H4** Nurses who assess a low risk for organism transmission (either in general on the unit, own risk of contributing to organism transmission or risk for oneself becoming infected at work) rate high levels of self-efficacy to medical asepsis.

## 2 Methods

### 2.1 Study design and setting

This study was cross-sectional and used a correlational design [25]. Data were collected from April to December 2019. A list of all surgical and orthopaedic units in Sweden providing 24-h care (N = 207) was established. Based on the assumption of getting a response rate of approximately 50%, we aimed to invite about 1,000 nurses for participation. This approach was considered to have the potential to generate a sample large enough to be representative. Forty-two units were therefore randomised from the list. Among the randomised units, 25 units located in 22 hospitals accepted participation. Since surgical site infections are one of the most frequently reported healthcare-associated infections, it was considered appropriate to include surgical and orthopaedic hospital units. A comprehensive description of the units’ characteristics is presented in Table 1.

**Table 1:** Characteristics of the included units

Hospital type	*n* = 25
District hospital	10
Community hospital	5
Regional/university hospital	9
Private hospital	1
**Unit speciality**
Surgical	15
Orthopaedic	10
**Number of patient beds**
10–19	5
20–29	17
30–39	2
40–49	1
**Entire unit open**
Yes	13
No (due to lack of personnel)	12
**Type of patient rooms**
Only single rooms	1
Single and double rooms	8
1–3 beds per room	3
1–4 beds per room	13
**Personnel education in hygiene guidelines**
Continuously/annually	15
At the start of employment	8
No	2
**FLMs’ estimation of levels of personnel turnover**
Low	15
High	10
**FLMs’ estimation of levels of patient overcrowding**
Low	13
High	12
**Placement of overcrowded patients**
In patient rooms	18
In the corridor	7
**FLMs’ estimation of overall patient-level workload**
Low need	4
Medium need	12
High need	9
**FLMs’ span of control**
20–39	7
40–59	12
60–79	6
**FLMs’ perceived conditions for the HCP to follow hygiene guidelines**
Good conditions	19
Poor conditions	6

Abbreviations: FLM First-line manager, HCP Healthcare personnel.

### 2.2 Sample and procedure

After the first-line managers had accepted participation for the respective unit, they shared a list of email addresses to nurses (registered nurses and licensed practical nurses) who met the inclusion criteria: they had to be working either full-time or part-time and, have permanent employment or be paid by the hour. Personnel who were not working at the time, e.g., because of parental leave or long-term sick leave, were excluded. The nurses received the study material at their workplace, either by regular post or email, depending on the first-line manager’s desire. For those who received the study material by regular post, this consisted of an informational letter, a coded questionnaire and a stamped return envelope. They could choose between returning the questionnaire by post or using the web link or QR code in the informational letter. Where the first-line managers preferred that the nurses receive the study material by email, this consisted of an informational letter, a link to the questionnaire and a personal code. Two reminders were sent by email to non-responders. In total, we asked 985 nurses to respond to the questionnaire, of whom 417 responded, resulting in a response rate of 42%. Participation was voluntary; participants could withdraw at any time, and confidentiality was ensured. Structured telephone interviews were performed with the respective first-line managers to gather information about each unit’s characteristics, e.g., managers’ span of control, number of patient beds and type of patient rooms.

### 2.3 Measures

The questionnaire opened with questions on demographics (e.g., age, gender and education) and professional characteristics (e.g., years of work experience and working time). Additionally, three study-specific questions (A–C) concerning assessment of risks for organism transmission at work were included: A. How do you assess the risk for organism transmission at your workplace? B. How do you assess the risk that you contribute to organism transmission at your workplace? C. How do you assess your risk of getting infected during a work day? Items were rated on a five-point scale from 1 (low risk) to 5 (high risk). This was followed by the four questionnaires described below.

#### 2.3.1 Self-efficacy to medical asepsis in care situations

Self-efficacy to medical asepsis in care situations was assessed using the Infection Prevention Appraisal Scale (IPAS). Since there was no previous questionnaire focusing on self-efficacy to medical asepsis, two researchers in the research group developed this questionnaire based on Bandura’s self-efficacy theory [10] and its associated guide for instrument development [26]. The questionnaire consists of 15 items regarding the respondent’s perception of self-efficacy to medical asepsis and general and specific hygiene principles [8]. The principles covered five aspects: work clothes (3 items), disinfection (4 items), glove usage (3 items), aseptic technique (3 items) and jewellery/nails (2 items). It is preliminarily confirmed as unidimensional (by using parallel analysis on unpublished data from registered nurses and licensed practical nurses at medical units). Responses are given on an eleven-point scale from 0 (not sure at all) to 10 (totally sure). The items are summed up to generate a total score. Face validity [25] was assessed with ten registered nurses and licensed practical nurses, and minor linguistic adjustments were made. Their responses were not included in further analyses. Item and scale content validity index [25] were shown to be excellent as rated by ten independent infection prevention nurses (unpublished data). Cronbach’s alpha was 0.82 in the present study.

#### 2.3.2 Structural empowerment

Structural empowerment was measured using the Conditions of Work Effectiveness Questionnaire-II (CWEQ-II) [16], which has been translated into Swedish [27]. The CWEQ-II consists of 19 items measuring six factors of structural empowerment: access to opportunity, resources, information, support, formal power and informal power. Items are rated on a five-point scale from 1 (none) to 5 (a lot). Higher scores represent stronger perceptions of working in an empowered environment. In addition, two items measure ‘global empowerment’, which is a validation index (mean of the sum of the two items). Factor scores are averaged, and either the first four subscales or all six subscales are then summed up to give a total score. The six-subscale version was used in this study. A total score of 6–13 implies low levels of empowerment, 14–22 moderate levels and 23–30 high levels [28]. In this study, Cronbach’s alpha ranged from 0.71 to 0.86 within the subscales and the total Cronbach’s alpha score was 0.79, which is similar to those of previous studies [29, 30].

#### 2.3.3 Work engagement

Work engagement was assessed using the 9-item Utrecht Work Engagement Scale (UWES-9) [31]. The Swedish version, which has been confirmed to have acceptable validity and reliability [32], was used. The instrument includes the three dimensions of vigour, dedication and absorption, with three items each. Recent studies have revealed one factor to be appropriate [33, 34] and this has therefore been used in this study. Items are rated on a seven-point scale from 0 (never) to 6 (always). Items were summed and divided by the number of items. Higher scores represent higher overall work engagement. A total mean score≤1.77 represents very low work engagement, 1.78–2.88 low, 2.89–4.66 average, 4.67–5.50 high and≥5.51 represents very high work engagement [35]. Cronbach’s alpha in this study was 0.93.

#### 2.3.4 Work-related stress

Work-related stress was measured using the United Kingdom Health & Safety Executive (HSE) Management Standards Indicator Tool [36]. The tool is published by the British authority of health prevention and safety at work and consists of 35 items measuring six primary stressors: control, demands, role, change, relationships and support (which is further divided into the subscales Manager support and Colleague support). Responses are given on a five-point scale from 1 (poor) to 5 (desirable), measuring how well the employer is performing in managing each of the six work-related stressors in relation to the management standards [24]. The instrument is frequently used and has confirmed acceptable validity and reliability [37]. Permission to translate the instrument was obtained, and it was translated into Swedish using a backward-forward translation technique, inspired by Beaton’s guidelines [38]. In the first step, a bilingual expert translated the instrument to Swedish. Then, it was presented to a small group (*n* = 5) of academy staff/registered nurses to control items in terms of relevance, the scoring of each question, clarity and fluency. It was apparent from their responses that the Swedish version was understandable, and there were no suggestions for changing the wording or rephrasing any of the questions. A second bilingual expert obtained a blinded back-translation, and a final agreement was achieved. The answers from the academy staff/registered nurses in the face validity were not included in further analyses. Factors were summed up and divided by the number of factors. Participants’ scores are compared with benchmark scores that are expressed in percentiles in different colours, to facilitate interpretation of the results. Results below the 20th percentile are marked red and indicate that urgent action is needed. Scores below the 50th but above the 20th percentile are yellow, meaning that improvements are needed. Results above the 50th and below the 80th percentile are aqua, meaning that performance is good, but with potential for improvement, and scores above the 80th percentile are green, indicating good results, with a need to maintain performance [39]. Cronbach’s alpha ranged from 0.78 to 0.91 in this study and the total Cronbach’s alpha value was 0.82.

### 2.4 Data analysis

Data analysis was performed using IBM SPSS Statistics for Windows, Version 27.0 (IBM Corp., Armonk, NY, USA). Descriptive statistics were calculated. As a precondition for the correlation analyses, we tested whether the variables were normally distributed. The majority were not and Spearman’s rho for bivariate correlation was therefore calculated to examine correlations between variables. Missing values for items varied from 0.5 to 3% and how they were handled depended on the instrument. For IPAS, missing values were replaced with the median value of each item. For UWES-9, the mean value for each participant was calculated and replaced the missing value. For HSE and CWEQ-II, the participants had to answer all the questions for each variable; otherwise, there would be over 10% missing. If they did not, the factor was removed, and we used pairwise deletion in the analyses, since this is recommended for correlational analyses [40]. In the interpretation of correlational coefficients, we were guided by Guilford, who describes values less than 0.20 as slight, almost negligible relationship; 0.20–0.40 low correlation, a definite, but small relationship; 0.40–0.70 moderate correlation, a substantial relationship; 0.70–0.90 high correlation, a marked relationship; and 0.90–1.00 very high correlation, a very dependable relationship [41].

The Kruskal-Wallis H non-parametric test was used to compare self-efficacy to medical asepsis with the working condition variables. Before the analyses, we grouped UWES-9 scores into three categories: Low (which included Very low and Low), Medium (which included Very high and Medium), and High (which included Very high and High); the score average was maintained. We also grouped the scores from the answers concerning assessed risks for organism transmission (questions A–C), resulting in three groups: Low (which includes Low and Medium/low) and High (including Medium/high and High); the score Medium was maintained. In all analyses, *p*-values below 0.05 (two-tailed) were regarded as statistically significant. Internal consistency was measured with Cronbach’s alpha, which demonstrated acceptable values (*α*> 0.70) for all study questionnaires.

## 3 Results

### 3.1 Sample characteristics

Nine of ten nurses were female. The mean age of participants was 40.5 years (SD = 13.9, range 19–67) and there was an equal distribution between registered nurses and licensed practical nurses. The mean years of work experience in the current work role was 13.5 years (SD 12.9), and the mean time at the present unit was 7.8 years (SD 9.0). The majority had their education in Sweden, and nine participants were educated in another country (Bosnia, Croatia, Finland, Lithuania, Philippines, Poland, Uganda, United States and the Netherlands). A comprehensive description of the nurses is presented in Table 2.

**Table 2:** Characteristics of the included nurses

Healthcare personnel	*n* = 417 (%)
**Age (years), mean (SD)**	40.5 (13.9)
**Years of work experience, mean (SD)**	13.5 (12.9)
**Years at present unit, mean (SD)**	7.8 (9.0)
**Gender**
Female	378 (91.1)
Male	37 (8.9)
**Education**
Assistant nurse	197 (47.2)
Registered nurse	204 (48.9)
Nurse specialist	16 (3.8)
**Country of education**
Sweden	402 (97.8)
Other	9 (2.2)
**Working time**
Full-time	307 (75.2)
Part-time	101 (24.8)
**Work shift**
Day	32 (7.7)
Day/evening	246 (59.7)
Night	51 (12.3)
Rotational work	84 (20.3)

Abbreviations: SD standard deviation. When totals do not add up to 417, there are missing internal data.

### 3.2 Self-efficacy to medical asepsis in care situations

The nurses rated high levels of self-efficacy to medical asepsis in care situations, with a total mean score of 137.1 (SD = 12.4, Min = 82, Max = 150), see Table 3. The nurses scored lowest confidence regarding the item *Always use gloves when drawing blood* (M = 8.1, SD = 2.8, Min = 1, Max = 10). The item with highest confidence (M = 9.9, SD = 0.2, Min = 7, Max = 10) was *Never forget to take off my wrist watch before starting work.*

**Table 3:** Descriptive statistics and bivariate correlations (Spearman’s rho) between study variables

Variable	Scale range	Min–Max	Mean	SD	Median	IQR	Skewness	Kurtosis	1. Sum IPAS	2. Mean UWES	3. Sum CWEQ	4. Mean HSE	5. Risk-A	6. Risk-B
1. IPAS (sum)	0–150	82–150	137.1	12.4	140	13	–1.801	3.975
2. UWES-9 (mean)	0–6	1–6	4.7	0.9	4.9	1.2	–1.045	1.446	0.268**
3. CWEQ-II (sum)	6–30	8–30	20.4	3.7	20.1	5	–0.045	0.092	0.255**	0.546**
4. HSE (mean)	1–5	2.0–4.8	3.8	0.4	3.8	0.6	–0.463	0.999	0.254**	0.457**	0.692**
5. Risk-A	1–5	1–5	2.6	1.1	3	1	0.443	–0.048	–0.195**	–0.232**	–0.220**	–0.240**
6. Risk-B	1–5	1–5	1.9	0.9	2	1	0.896	0.654	–0.204**	–0.224**	–0.130*	–0.210**	0.578**
7. Risk-C	1–5	1–5	1.9	0.9	2	1	0.757	–0.011	0.008	–0.207**	–0.190**	–0.279**	0.558**	0.583**

Abbreviations: SD Standard deviation, IQR Interquartile range, IPAS Infection Prevention Appraisal Scale, UWES-9 Utrecht Work Engagement Scale, CWEQ-11 Conditions of Work Effectiveness Questionnaire, HSE Health & Safety Executive Management Standards Indicator Tool. Risk-A How do you assess the risk for organism transmission at your workplace? Risk-B How do you assess the risk that you contribute to organism transmission at your workplace? Risk-C How do you assess your risk of getting infected during a work day? **Correlation is significant at the level 0.01 (two-tailed).

### 3.3 Structural empowerment

Total rates of structural empowerment were moderate (M = 20.4, SD = 3.7, Min = 8, Max = 30). The subscale with the highest scores was Access to opportunity (M = 3.8, SD = 0.7, Min = 1, Max = 5) and the lowest was Access to information (M = 3.1, SD = 0.9, Min = 1, Max = 5). As a general empowerment measure, Global empowerment had a mean score of 3.3 (SD = 0.9, Min = 1, Max = 5), which is in line with the other subscales (Table 4). Correlational tests used the mean scores of the six subscales of CWEQ-II and revealed low correlation and a definite, but small relationship between structural empowerment and self-efficacy to medical asepsis (r_s_ = 0.255, *p* < 0.001), see Table 3. The highest correlation for the subscales in CWEQ-II and self-efficacy to medical asepsis was Access to support (r_s_ = 0.295, *p* < 0.001), see Table 4. The results from the Kruskal-Wallis H test confirmed that nurses who rated high levels of structural empowerment had significantly higher levels of self-efficacy to medical asepsis compared with the group with average structural empowerment. No statistically significant differences were found between the low and average groups. All results from the comparative analysis are shown in Table 5.

**Table 4:** Descriptive statistics and bivariate correlations (Spearman’s rho) between IPAS and respective factor in CWEQ and HSE

Factor	Scale range	Min–Max	Mean	SD	IPAS	CWEQ	1	2	3	4	5	6	7	HSE	8	9	10	11	12	13
**IPAS (sum)**	0–150	82–150	137.1	12.4
**CWEQ-II (sum)**	6–30	8–30	20.4	3.7	0.255**
1 Opportunity	1–5	1–5	3.8	0.7	0.144**	0.566**
2 Information	1–5	1–5	3.1	0.9	0.179**	0.635**	0.236**
3 Support	1–5	1–5	3.3	0.9	0.295**	0.756**	0.381**	0.381**
4 Resources	1–5	1.33–5	3.4	0.8	0.200**	0.669**	0.200**	0.366**	0.443**
5 Formal power	1–5	1–5	3.2	0.9	0.163**	0.814**	0.367**	0.480**	0.549**	0.506**
6 Informal power	1–5	1–5	3.7	0.8	0.100*	0.612**	0.382**	0.196**	0.352**	0.272**	0.434**
7 Global empowerment	1–5	1–5	3.3	0.9	0.195**	0.624**	0.304**	0.334**	0.377**	0.564**	0.618**	0.419**
**HSE (mean)**	1–5	2–4.8	3.8	0.4	0.254**	0.692**	0.313**	0.368**	0.569**	0.607**	0.588**	0.352**	0.646**
8 Demands	1–5	1–4.88	3.1	0.6	0.134**	0.247**	–0.34	0.189**	0.195**	0.536**	0.166**	–0.054	0.369**	0.576**
9 Control	1–5	1.17–5	3.2	0.6	0.171**	0.509**	0.224**	0.293**	0.315**	0.945**	0.502**	0.273**	0.556**	0.636**	0.349**
10 Manager support	1–5	1–5	3.8	0.8	0.217**	0.363**	0.347**	0.320**	0.580**	0.480**	0.556**	0.371**	0.498**	0.810**	0.312**	0.419**
11 Colleague support	1–5	2–5	4.2	0.5	0.114*	0.408**	0.194**	0.208**	0.361**	0.381**	0.352**	0.326**	0.417**	0.700**	0.312**	0.361**	0.565**
12 Relationships	1–5	1–5	4.1	0.6	0.003	0.327**	0.219**	0.126*	0.289**	0.286**	0.223**	0.167**	0.363**	0.578**	0.392**	0.174**	0.404**	0.434**
13 Role	1–5	2.2–5	4.2	0.5	0.217**	0.485**	0.305**	0.387**	0.390**	0.417**	0.394**	0.234**	0.425**	0.627**	0.298**	0.360**	0.452**	0.492**	0.257**
14 Change	1–5	1–5	3.4	0.8	0.232**	0.608**	0.316**	0.395**	0.485**	0.471**	0.545**	0.336**	0.512**	0.773**	0.239**	0.502**	0.659**	0.431**	0.308**	0.446**

Abbreviations: IPAS Infection Prevention Appraisal Scale, CWEQ-II Conditions of Work Effectiveness Questionnaire, HSE Health & Safety Executive Management Standards Indicator Tool, SD Standard deviation. *Correlation is significant at the level 0.05 (two-tailed). **Correlation is significant at the level 0.01 (two-tailed).

**Table 5:** Comparisons between self-efficacy to medical asepsis and grouped working condition variables

Working condition variables	Self-efficacy to medical asepsis	Percent within the sample
**Structural empowerment**
Scores: 6–13 Low empowerment, mean (SD) IQR	138.5 (7.3) 13	2.1
Scores: 14–22 Moderate empowerment, mean (SD) IQR	135.4 (13.6) 15	68.1
Scores: 23–30 High empowerment, mean (SD) IQR	140.4 (9.4) 10	29.8
Test statistics H (df)	12.136 (2)
*p*-value	**0.002**
Bonferroni *post-hoc* test	Moderate–low 1.000 **Moderate–high 0.002** Low–high 0.943
**Work engagement**
Scores:≤2.88 Low work engagement, mean (SD) IQR	133 (13.3) 23	2.9
Scores: 2.89–4.66 Average work engagement, mean (SD) IQR	134.1 (14.4) 14	35.2
Scores:≥4.67 High work engagement, mean (SD) IQR	138.9 (10.8) 13	61.9
Test statistics H (df)	16.249 (2)
*p*-value	**<0,000**
Bonferroni *post-hoc* test	Low–average 1.000 Low–high 0.237 **Average–high < 0.000**
**Work-related stress**
Scores: < 3.24 Red - Urgent action required mean (SD) IQR	132.9 (16.7) 17	13
Scores: 3.25–3.96 Yellow - Improvement needed mean (SD) IQR	135.8 (12.4) 14	54.2
Scores: 3.97–4.49 Aqua - Good performance, potential improvement mean (SD) IQR	139.8 (10.8) 12	28.6
Scores: > 4.50 Green - Doing well, maintain performance mean (SD) IQR	143.5 (6.5) 7	4.2
Test statistics H (df)	16.895 (3)
*p*-value	**0.001**
Bonferroni *post-hoc* test	Red–yellow 1.000 Red–aqua 0.062 **Red–green 0.029 Yellow–aqua 0.017 Yellow–green 0.031** Aqua–green 1.000
**General risk for organism transmission at work**
Scores: 1–2 Low risk, mean (SD) IQR	139.9 (9.3) 11	46.2
Scores: 3 Medium risk, mean (SD) IQR	135.4 (12.5) 16	39
Scores: 4–5 High risk, mean (SD) IQR	132.3 (17.9) 23	14.8
Test statistics H (df)	14.093 (2)
*p*-value	**0.001**
Bonferroni *post-hoc* test	High–medium 1.000 **High–low 0.027 Medium–low 0.002**
**Own risk to contribute to organism transmission at work**
Scores: 1–2 Low risk, mean (SD) IQR	138.2 (10.9) 13	76.5
Scores: 3 Medium risk, mean (SD) IQR	133.7 (14.9) 22	17.9
Scores: 4–5 High risk, mean (SD) IQR	130.7 (19.1) 22	5.6
Test statistics H (df)	6.591 (2)
*p*-value	**0.037**
Bonferroni *post-hoc* test	High–medium 1.000 High–low 0.302 Medium–low 0.095
**Risk of becoming infected oneself at work**
Scores: 1–2 Low risk, mean (SD) IQR	137.0 (11.7) 13	73.5
Scores: 3 Medium risk, mean (SD) IQR	136.9 (15.2) 15	20.2
Scores: 4–5 High risk, mean (SD) IQR	137.6 (12.7) 20	6.3
Test statistics H (df)	1.962 (2)
*p*-value	0.375

Abbreviations: SD Standard deviation. IQR: Interquartile range. df: Degrees of freedom. H: Kruskal-Wallis test. Significant values in bold.

### 3.4 Work engagement

Work engagement had a relatively high rating (M = 4.7, SD = 0.9, Min = 1, Max = 6), and a definite, but small positive relationship was found between self-efficacy to medical asepsis in care situations and work engagement (r_s_ = 0.268, *p* < 0.001), see Table 3. When comparing groups, the results revealed significantly higher self-efficacy to medical asepsis in the group rating high work engagement compared with the group rating average work engagement. The results revealed no significant differences between the groups that scored low and average (Table 5).

### 3.5 Work-related stress

Perceived overall work-related stress (M = 3.8, SD = 0.4, Min = 2, Max = 4.8) was rated as category yellow, i.e., improvement needed. The highest scores were in the subscales Colleague support and Role (M = 4.2, SD = 0.4, Min = 2, Max = 5) for each, which ended up in the category aqua, i.e. good performance with potential for improvement. The lowest score was on the subscale Demands (M = 3.1, SD = 0.6, Min = 1, Max = 4.9), ending up in the category red, i.e. urgent action required; see Tables 4 and 5. The correlational test revealed a definite, but small relationship between overall work-related stress and self-efficacy to medical asepsis in care situations (r_s_ = 0.254, *p* < 0.001), see Table 3. The highest correlation for the subscales in HSE and self-efficacy to medical asepsis was Change (r_s_ = 0.232, *p* < 0.001). No correlation was found between self-efficacy to medical asepsis and the subscale Relationships (r_s_ = 0.003), see Table 4. The Kruskal-Wallis H test revealed significant differences between the groups red and green, yellow and aqua, and yellow and green, see Table 5.

### 3.6 Assessment of risks for organism transmission at work

The results showed that the nurses assessed the general risk for organism transmission at work as medium–high (M = 2.6, SD = 1.1, Min = 1, Max = 5); see Risk-A in Table 3. The mean scores for both own risk of contributing to organism transmission (Risk-B in Table 3) and the risk for oneself becoming infected at work (Risk-C in Table 3) were as follows: (M = 1.9, SD = 0.9, Min = 1, Max = 5). A definite, but small negative relationship was found between self-efficacy to medical asepsis in care situations and the assessment of general risk for organism transmission (r_s_ = –0.195, *p* < 0.001) and the assessment of own risk of contributing to organism transmission at the workplace (r_s_ = –0.204, *p* < 0.001). There was no correlation between risk assessments for becoming infected oneself and self-efficacy to medical asepsis (*r* = 0.008), see Table 3. The comparative analysis revealed significant values regarding general risk and self-efficacy to medical asepsis in care situations between the groups high–low and medium–low. No significant relationships were found between self-efficacy to medical asepsis and the assessed own risk of contributing to organism transmission or becoming infected at work (see Table 5).

## 4 Discussion

This study revealed that nurses rated high levels of self-efficacy to medical asepsis in care situations and a definite, but small relationship was found between the working conditions of nurses and self-efficacy to medical asepsis. Self-efficacy is described as a person’s belief in their ability to succeed in different situations [10] and has been found to influence employees’ performance at work [12]. Worldwide, registered nurses are describing that they are experiencing undesirable working conditions [4], and the association between nurses’ working conditions and patient safety is well known [42–45]. Moreover, healthcare-associated infections can be the consequence of deficient medical asepsis in care situations and non-compliance with hygiene principles. Though our results revealed that nurses rated high levels of self-efficacy to medical asepsis in care situations, this does not necessarily correspond to the actual performance of medical asepsis and compliance with hygiene principles. Past research has found that healthcare personnel often overestimate their hand hygiene performance in relation to observed behaviour [46–48] and that nurses are often unaware of performed risk behaviours for organism transmission [48].

Healthcare-associated infections also include occupational infections [49]. In this study, there was no relationship between assessment of risk for oneself becoming infected at work and self-efficacy to medical asepsis. This could imply that nurses primarily associate medical asepsis and compliance to hygiene principles with patient safety rather than occupational infections. The fact that nurses assessed the risk for oneself becoming infected at work as low can also imply that nurses use hygiene principles, such as protective clothing and gloves, to protect themselves rather than patients, which has been found in previous research [46]. Furthermore, the nurses assessed the general risk for organism transmission as higher than the risk of themselves contributing to organism transmission at work. This is also in line with previous research, showing that nurses often rate their own ability and compliance with hygiene principles more highly than that of colleagues [46].

Regarding structural empowerment, most nurses rated moderate levels, and the hypothesis that nurses who rate high levels of structural empowerment would also rated high levels of self-efficacy to medical asepsis could partly be supported. Structural empowerment has in previous studies been found to positively influence both work effectiveness and patient safety [5]. Structural empowerment implies, among other things, having access to information and resources [15]. As previously described, a systematic qualitative literature review found that healthcare personnel’s perceptions of their work environment, e.g., access to resources and information, influenced compliance with hygiene principles. Accordingly, when employees are empowered in their jobs, it increases their compliance with hand hygiene guidelines [17], and is thus important also for patient safety during care.

In this study, the majority of nurses rated a high level of work engagement. The hypothesis that nurses who rate high levels of work engagement would also rate high levels of self-efficacy to medical asepsis was partly supported, with significant differences between nurses who rated high versus average work engagement. Nurses have expressed that psychosocial working environments, such as colleagues’ and managers’ engagement and the workplace culture regarding infection prevention, influence their infection prevention behaviour [48]. Since employees with a high level of work engagement often experience more positive emotions and enthusiasm and have the ability to transfer their engagement to others, it is important to create a workplace that increases and maintains work engagement among the personnel [50].

Regarding work-related stress, more than 65% of the nurses in this study gave answers indicating that improvement was needed, of which 13% required urgent action. Significant differences were found between self-efficacy to medical asepsis in care situations and several groups regarding work-related stress. Thus, the hypothesis proposing that nurses who rated low levels of work-related stress would rate high levels of self-efficacy to medical asepsis was supported. Work-related stress is a common difficulty for healthcare personnel [7, 21] and high levels of work-related stress have been found to increase the risk for healthcare-associated infections and negatively impact healthcare personnel’s compliance with medical asepsis routines [51]. Work-related stress has also been discussed by nurses as a reason for non-compliance with hygiene guidelines [48]. Previous research has found that work-related stress can be predicted by several psychosocial workplace factors such as high job demands, lack of support, insufficient interpersonal relations and the work role [52], i.e., factors included in the theory of structural empowerment. In this study, we found work-related stress to have a relationship to structural empowerment among nurses. However, qualitative studies investigating nurses’ experiences of reasons for work-related stress are scarce, and this phenomenon should be studied in greater detail and taken into account when designing future qualitative studies aiming to investigate this topic further.

This study’s results confirmed that nurses experiencing high access to structural empowerment, high work engagement and low levels of work-related stress assess higher levels of self-efficacy to medical asepsis in care situations, as definite, but small relationships between variables were found. Still, it is difficult to conclude to what extent self-efficacy to medical asepsis is related to nurse’s working conditions, as the relationship might be non-linear. The nurses in this study rated the highest and the most positive scores concerning work-related stress in the subscale Colleague support. Colleague support can be connected to element number 3 in Bandura’s theory of self-efficacy. This element concerns verbal persuasion and implies that people are coached by others to strengthen belief in their personal capacity. It can also be connected to element number 4, that psychological states influence belief in capability [10]. Regarding structural empowerment, the highest correlation to self-efficacy was found in the subscale Access to support, which further strengthens this connection.

According to the theory, self-efficacy relates to a person’s belief in their ability and consequently affects human behaviour [10]. Past research has pronounced that self-efficacy appears to impact the effectiveness of interventions to improve hand hygiene behaviour, but that more research is needed [14]. Based on our results, we agree that further investigations are needed to determine potential relationships and to what extent the various factors intertwine. As a suggestion, this could be done by including instruments focusing on other working conditions, such as working climate, but also using a qualitative perspective, as proposed earlier.

### 4.1 Limitations

An overall limitation was the cross-sectional design, which does not make it possible to find causal relationships between outcomes. In the correlational analysis, we found a low correlation and a definite, but small relationship between nurses’ working conditions and self-efficacy to medical asepsis. However, based on these results, we cannot conclude which variables affect what. The assumptions for multiple linear regression analyses were not met, since this was not possible with our available data, which is another limitation.

Since the first-line managers could choose how the participants would receive the questionnaire and since we did not request a response confirmation, we cannot guarantee that all potential participants received the information, which might have affected the response rate.

The Infection Prevention Appraisal Scale is new and was developed because there was no previous questionnaire that measured self-efficacy to medical asepsis in care situations. However, the questionnaire is subject-specific in line with the theory and its associated guide for instrument development and both the item and scale content validity index was rated as excellent by ten independent infection prevention nurses, in not yet published data. This study is the first to investigate whether self-efficacy relates to nurses’ working conditions and further psychometric tests are required regarding the Infection Prevention Appraisal Scale. The questions concerning the assessment of risks for organism transmission (A–C) are study-specific and were included to capture the nurses’ own assessments of these risks at work. Therefore, validity and reliability tests are lacking, which is a potential limitation [25].

## 5 Conclusions

This study revealed that nurses rated high levels of self-efficacy to medical asepsis in care situations, and to some extent, this seemed to have a relationship to structural empowerment, work engagement and work-related stress, i.e., hypotheses 1–3 were partly supported. Hypothesis 4, suggesting that nurses who assessed a low risk for organism transmission would rate high levels of self-efficacy to medical asepsis, was supported when it came to general risk and own risk of contributing to organism transmission, but not the risk for oneself becoming infected at work. To conclude, we can see that there was some kind of relationship between nurses’ self-efficacy to medical asepsis and their working conditions, but more research is needed. Until we know more about how these factors are associated, it would be valuable if nursing management works to promote adequate working conditions and high levels of self-efficacy among nurses, which can be beneficial for both them and the patients they care for.

## Ethical approval

This study was conducted in accordance with applicable ethical rules and national laws. The Swedish ethical review authority approved the study protocol (reg. no. 2019-00530).

## Informed consent

Participation was strictly voluntary and participants could withdraw at any time without giving a reason. Confidentiality was ensured and informed consent was obtained from all participants.

## Conflict of interest

The authors declare that they have no conflict of interests.
